# Two new species of the genus *Acrolepiopsis* Gaedike, 1970 (Lepidoptera, Glyphipterigidae) from Korea

**DOI:** 10.3897/BDJ.13.e163874

**Published:** 2025-09-11

**Authors:** June-Hyeok Jeong, Un-Hong Heo, Ji-Young Lee, Jae-In Oh, Young-Gwang Song, Sang-Yoon Kim, Yonghwan Park, Bong-Kyu Byun

**Affiliations:** 1 The Science Museum of Natural Enemies, Geochang, Republic of Korea The Science Museum of Natural Enemies Geochang Republic of Korea; 2 Godeok Lotte Castle Venerouche, Seoul, Republic of Korea Godeok Lotte Castle Venerouche Seoul Republic of Korea; 3 Hannam University, Daejeon, Republic of Korea Hannam University Daejeon Republic of Korea; 4 Jeonbuk National University, Jeonju, Republic of Korea Jeonbuk National University Jeonju Republic of Korea; 5 National Institute of Forest Science, Seoul, Republic of Korea National Institute of Forest Science Seoul Republic of Korea

**Keywords:** Lepidoptera, Glyphipterigidae, *Acrolepiopsis*, new species, DNA barcode

## Abstract

**Background:**

The family Glyphipterigidae comprises small-sized moths with a wingspan ranging from 8 to 14 mm. This family is mainly distributed in the Palaearctic and Oriental Regions. In Korea, five species of the genus *Acrolepiopsis* Gaedike, 1970 have been recorded. However, species-level identification within this genus is often challenging due to high morphological similarity amongst species.

**New information:**

In this study, two new species of the genus *Acrolepiopsis*, *A. quinquelobatae ***sp. nov.** and *A. koreana* **sp. nov.**, are described from Korea. Species delimitation is supported by COI gene analysis and morphological characteristics of genitalia, both of which reveal clear genetic divergence from other *Acrolepiopsis* species previously recorded in Korea. Photographs of adults and genitalia, along with information on host plants, are also provided.

## Introduction

Genus *Acrolepiopsis* (Lepidoptera, Glyphipterigidae, Acrolepiinae) is distributed worldwide, except for Australia and Oceania, with 37 species currently recognised (Gaedike 1997). In comparison to the nine species reported from geographically adjacent Japan (Yasuda 2013), only five species have been recorded in Korea (Sohn et al. 2018), indicating the potential for discovering additional species. Based on this observation, the present study was conducted.

The adults are small, with a wingspan of 11-14 mm. During the larval stage, they feed on the stems or leaves of plants in the families Liliaceae (such as onions, garlic, and Chinese yam) and Dioscoreaceae, earning them the common name "leek moth" (Medvedev 1990, Landry 2007, Kim et al. 2013, Sohn et al. 2018). Morphologically, the head is covered with dense scales, the labial palpus is large and strongly curved, and the forewings have a yellowish-brown or dark brown ground colour, marked with brown or whitish spots (Shin et al. 1983, Medvedev 1990).

This study is aimed to describe two new species of *Acrolepiopsis* Gaedike from Korea: *A. quinquelobatae *sp. nov. and *A. koreana* sp. nov.

## Materials and methods

All specimens examined in this study were collected either as larvae during the daytime or as adults at night using a light trap. Adult photographs were taken with a digital camera (Canon EOS 550D; Canon, Tokyo, Japan). Genitalia were conducted following the method described by Holloway (Holloway et al. 1987), and images were captured using a digital camera attached to a microscope (Leica M205C; Leica Microsystems, Wetzlar, Hesse, Germany). Type materials are deposited in the National Institute of Biological Resources(NIBR), Incheon, Korea and Systematic Entomology Lab., Hannam University (HNUSEL), Daejeon, Korea. All voucher specimens, used in the analysis for DNA barcoding, are deposited in HNUSEL.

DNA analysis was conducted using the hind legs of dried specimens of the new species and recorded species (Table 1). Genomic DNA was extracted using the DNeasy Blood & Tissue Kit (QIAGEN, Germany) following the manufacturer's protocol.

The CO1 gene was amplified using a SimpliAmp Thermal Cycler (Life Technologies Holdings Pte Ltd., Singapore) with the primers LepF1 (ATTCAACCAATCATAAAGATATTGG) and LepR1 (TAAACTTCTGGATGTCCAAAAAATCA) (Hebert et al. 2004). The PCR conditions followed the protocol provided by the Biodiversity Institute of Ontario, University of Guelph (https://ccdb.ca/).

DNA sequencing was performed by Macrogen, Inc. (Seoul, Korea), and the sequences were edited using Geneious Prime (version 2022.2.2, Geneious, Auckland, New Zealand). The finalised sequences have been uploaded to the GenBank database. The aligned sequences were analysed using the Neighbour-Joining (NJ) method in MEGA version 10.2.6 (MEGA, Pennsylvania State University, University Park, PA, USA). The Kimura 2-parameter model was applied, and the NJ tree was constructed with 1,000 bootstrap replications.

The abbreviations used in this study are as follows: GW (Gangwon-do), HNUSEL (Department of Biological Science & Biotechnology, Hannam University, Daejeon, Korea), NIBR(National Institute of Biological Resources).

## Taxon treatments

### Acrolepiopsis quinquelobatae

Jeong & Byun sp. nov.

96B48B68-5A92-50D6-909B-8E329020A18B

8B09C67A-0DB1-4C9A-8416-AECC12637FC2

#### Materials

- **Type status:** Holotype. **Occurrence:** catalogNumber: HNU_DNA_10049; recordedBy: UH Heo; individualCount: 1; sex: male; lifeStage: adult; otherCatalogNumbers: gen. slide no. HNUSEL-1297, DNA barcode Accession number (GenBank) PV748140; occurrenceID: 1D175540-5FA9-501B-9E6F-487D20686876; **Taxon:** order: Lepidoptera; family: Glyphipterigidae; genus: *Acrolepiopsis*; **Location:** country: Korea; stateProvince: GW; locality: Chuncheon-si, Mt. Geumbyeong; **Event:** year: 2023; month: vii; day: 28
- **Type status:** Paratype. **Occurrence:** catalogNumber: HNU_DNA_10048, 10050, 10051, 10052; recordedBy: UH Heo; individualCount: 4 ; sex: 1female, 3exs(abdomens missing); lifeStage: adult; otherCatalogNumbers: gen. slide no. HNUSEL-1295, DNA barcode Accession number (GenBank) PV748138, PV748139, PV748141, PV748142.; **Taxon:** order: Lepidoptera; family: Glyphipterigidae ; genus: *Acrolepiopsis*; **Location:** country: Korea; stateProvince: GW ; locality: Chuncheon-si, Mt. Geumbyeong; **Event:** year: 2023; month: vii ; day: 28

#### Description

**Adult** (Fig. 2a). Wingspan 8.0-9.0 mm.

**Head: **Covered with a dense patch of bright ochreous scales; antennae light brown, with scattered black rings; labial palpus straight, black dorsally, and brown ventrally.

**Thorax:** Black, mixed with dark brown scales; tegular covered with black scales. Legs dark silvery-white; fore- and hind tibia deep fuscous, and white ring in middle; hind tarsus black with alternating white bands. Fore-wing ground colour black, scattered with irregular light brown spots and several small white spots along margins; gradually darkened to deep black towards the apex. Costal streaks short yellow-ochreous spots, with 5–6 evenly spaced along the middle of outer margin; dorsal streaks located at 1/3 of hind margin, twice as long and thick as costal streaks, forming white triangular shapes. Hind-wings light grey, lanceolate, and gradually tapering towards tip.

**Abdomen:** Brown with a light grey colour.

**Male genitalia **(Fig. 3a)**.** Anal tube thick, membranous, and elongated arch-shape. Anellus has a sclerotised edge, forming an arch that gradually tapers towards tip, and half the length of anal tube. Valva narrows towards tip with slight curve, 7-8 long setae in middle of inner edge and short setae covering the tip. Sacculus rounded, swollen, covered halfway with long setae as long as valva. Saccus 1.7 times longer than valva, gradually tapering and becoming rounded towards tip, and surrounded by membranous structure. Aedeagus as long as the entire genitalia, gradually narrowing from 1/6 point and maintaining same thickness from 2/5 point to tip.

**Female genitalia **(Fig. 3b)**.** Apophyses anteriores and apophyses posteriores equal in length. Apophyses posteriores curve once at 1/3 point and again at the 2/3 point. Antrum flat, and sclerotised border. Ostium bursae narrow, circular shape with a sclerotised edge. Ductus bursae twice as long as apophyses posteriores, thick and sclerotised for first half, while remaining portion thin and membranous. Corpus bursae twice as long as apophyses anteriores, thin, membranous oval shape, with 2/3 region being thickened and wrinkled. Signa absent.

#### Diagnosis

This species is very similar to *Acrolepiopsis nagaimo* Yasuda, 2000, but it can be distinguished by the more pointed fore-wings and yellowish-brown costal streaks, as well as by the male genitalia, which have a longer and more slender aedeagus. (Yasuda 2000).

#### Etymology

The specific name *quinquelobatae* is derived from the genitive form of the plant name *Dioscorea quinquelobata*, indicating the host plant from which this species was reared.

#### Distribution

Korea (endemic).

#### Notes

**Figure 1.**
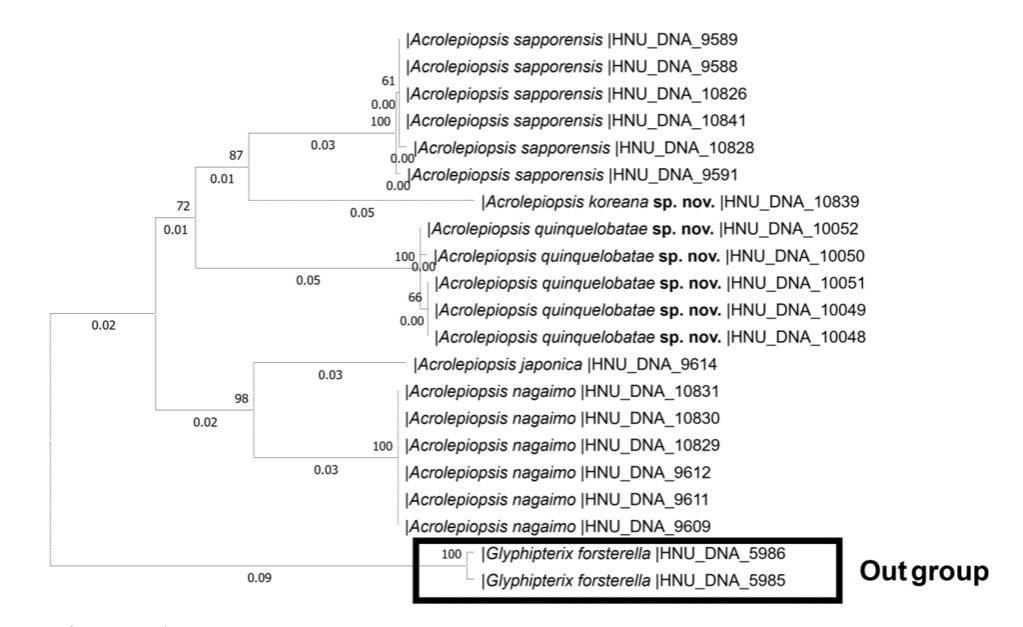
Neighbour–Joining tree of the genus *Acrolepiopsis* in Korea, based on the DNA barcode region.

This new species, *Acrolepiopsis quinquelobatae *sp. nov., shows a distinct genetic divergence from the closely-related Korean species *A. nagaimo*, with a genetic distance ranging from 9.0-10.8% (Fig. 1). In addition, a comparison with *A. orchidophaga*, which has a similar genital morphology, reveals that the male genitalia of *A. quinquelobatae* has a more slender valva and saccus, and the female genitalia is distinguished by a circular-shaped ostium bursae. The larva was reared on *Dioscorea quinquelobata* Thunb.

#### Host plant

The holotype and paratypes were obtained by rearing larvae on the host plant, *Dioscorea quinquelobata* Thunb. [Dioscoreaceae].

### Acrolepiopsis koreana

Jeong & Byun sp. nov.

3F7713B0-384C-58A2-B6D6-637C4DF55844

D74B529C-1E58-4A32-A4B2-CA0E3C41484D

#### Materials

- **Type status:** Holotype. **Occurrence:** catalogNumber: HNU_DNA_10839; recordedBy: BK Byun; individualCount: 1; sex: male; lifeStage: adult; otherCatalogNumbers: gen. slide no. HNUSEL-1009, DNA barcode Accession number (GenBank) PV748135.; **Taxon:** order: Lepidoptera; family: Glyphipterigidae; genus: *Acrolepiopsis*; **Location:** country: Korea; stateProvince: GW; locality: Jeongseon-gun; **Event:** year: 2024; month: viii; day: 25

#### Description

**Adult** (Fig. 2b). Wingspan 10 mm.

**Head: **Covered with a dense dark grey scales; antennae dark grey; labial palpus black, sharply curved upwards, and blunt tip.

**Thorax:** Silvery-brown with yellowish colour; tegula densely covered with dark black scales. Legs dark yellow; dorsal of fore- and hind tibia being black; hind tarsus black dorsally, with last segment yellowish-brown. Fore-wing ground colour dark grey with ochreous colour, gradually light brown towards apex; scattered with bright brown spots; costal streaks faint, short yellowish-brown spots, with 4–5 evenly spaced along margin; Dorsal streaks located at the 1/3 of hind margin, three times longer than costal streaks, light grey, and hook-shaped. Hind-wings light grey, lanceolate, and sharply tapering towards the tip.

**Abdomen:** Light grey with scattered dark colour.

**Male genitalia**. Unknown.

**Female genitalia **(Fig. 3c)**.** Apophyses anteriores and apophyses posteriores equal in length. Apophyses posteriores slightly curved at the 1/5 point. Ostium bursae round with slightly raised edge, surrounded by semicircular membranous part. Ductus bursae 1.5 times longer than apophyses posteriores, thick, sclerotised for 2/3 of its length and remaining portion being thin and membranous. Corpus bursae 1.3 times longer than ductus bursae, thin, membranous oval shape, with junction to the ductus bursae being thickened and wrinkled. Signa absent.

#### Diagnosis

This species is similar to *Acrolepiopsis quinquelobatae* sp. nov., but can be distinguished by light grey costal streaks and more extensively sclerotised region in the ductus bursae of the female genitalia.

#### Etymology

The species name of the new species was derived from the type locality (Korea).

#### Distribution

Korea (endemic).

#### Notes

This new species, *Acrolepiopsis koreana* sp. nov., shows a distinct genetic divergence of 8% from the closely-related Korean species *A. sapporensis*. In addition, a morphological comparison with *A. postomacula*, a species recorded from Korea and known as a genus hosta feeder, reveals that the adult of *A. koreana* sp. nov. has a generally darker grey ground colour on the hind-wing, and that the sclerotised portion of the ductus bursae in the female genitalia is shorter, occupying approximately 1/3 of its total length. Although the description is based on a single female holotype, we propose it as a new species because of its significant genetic divergence from closely-related species (Fig. 1).

## Supplementary Material

XML Treatment for Acrolepiopsis quinquelobatae

XML Treatment for Acrolepiopsis koreana

## Figures and Tables

**Figure 2a.**
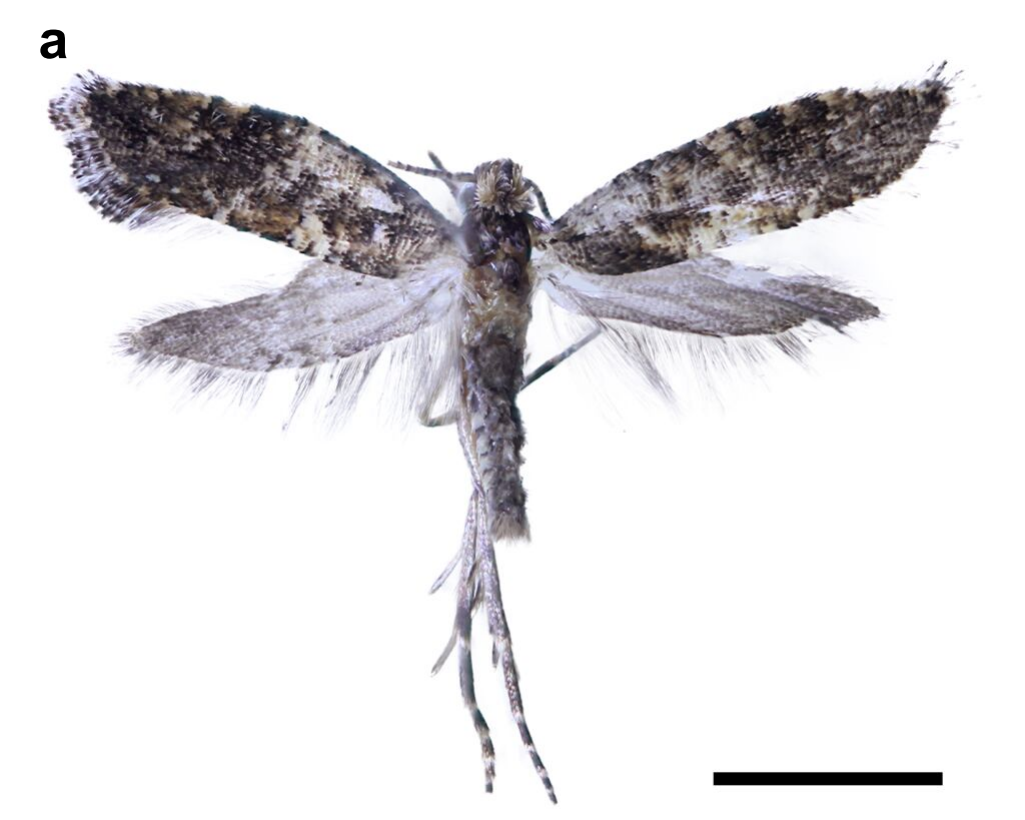
Adult of *A.* *quinquelobatae* sp. nov. (Holotype);

**Figure 2b.**
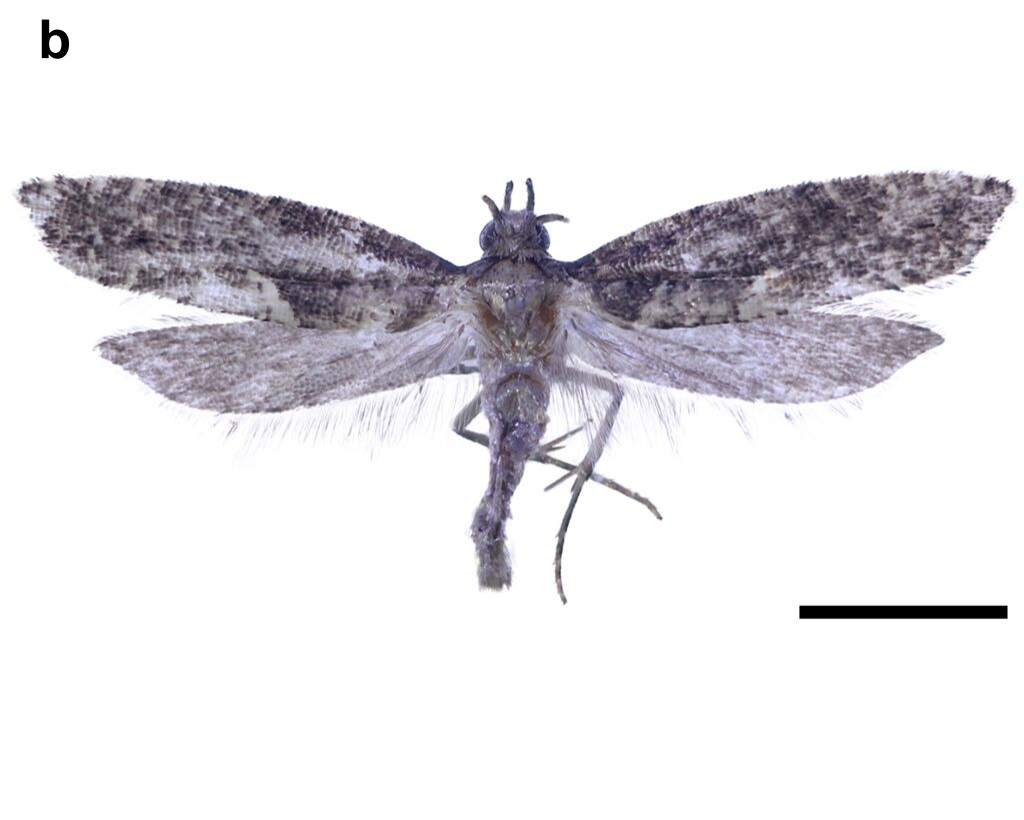
Adult of *A. koreana* sp. nov. (Holotype).

**Figure 3a.**
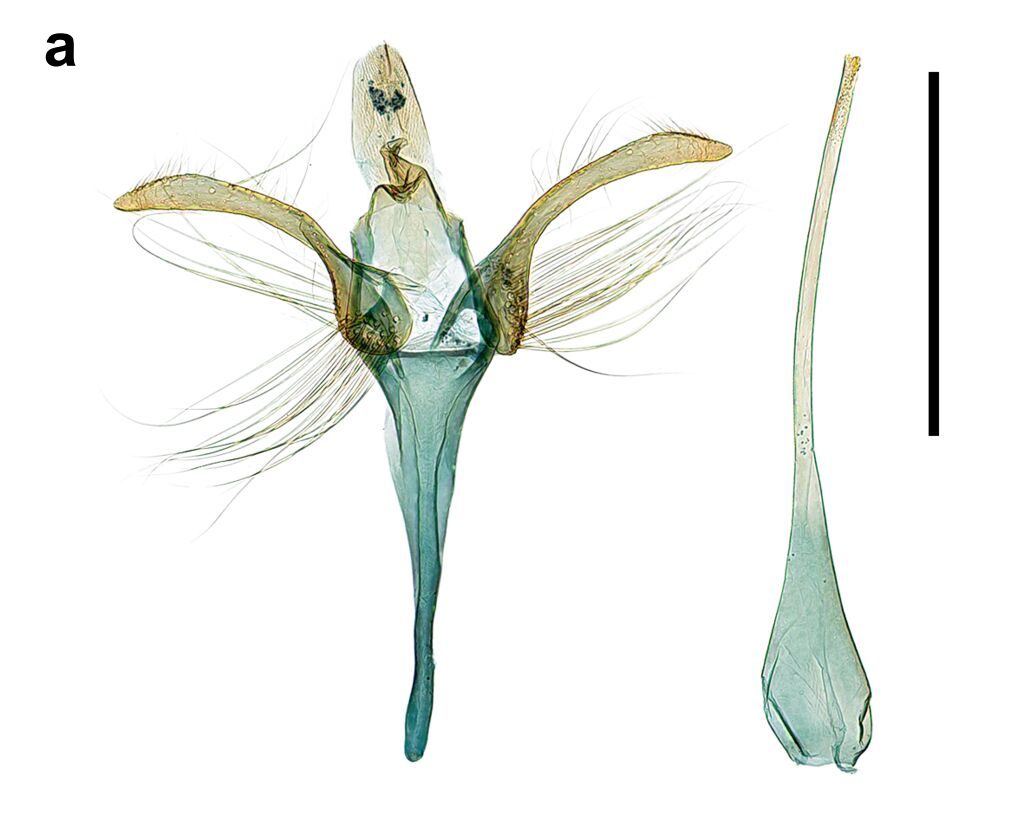
*A. quinquelobatae* sp. nov. (♂, Holotype, gen. slide no. HNUSEL-1297);

**Figure 3b.**
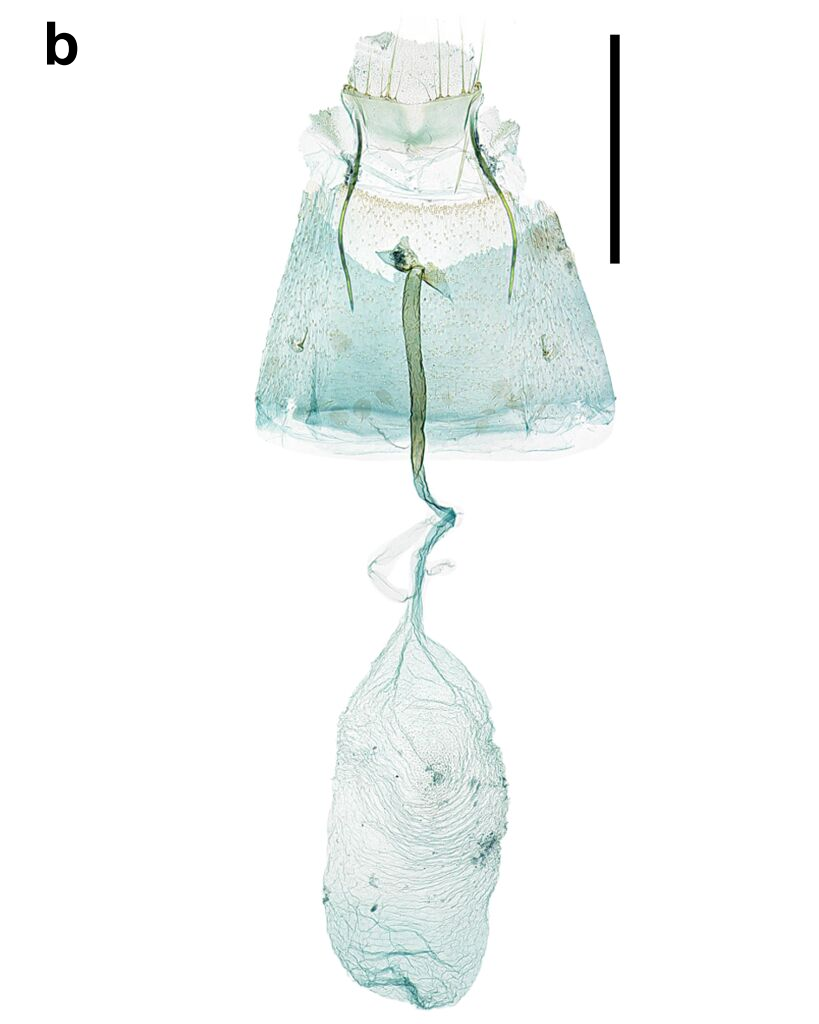
*A. quinquelobatae* sp. nov., female (♀, gen. slide no. HNUSEL-1295);

**Figure 3c.**
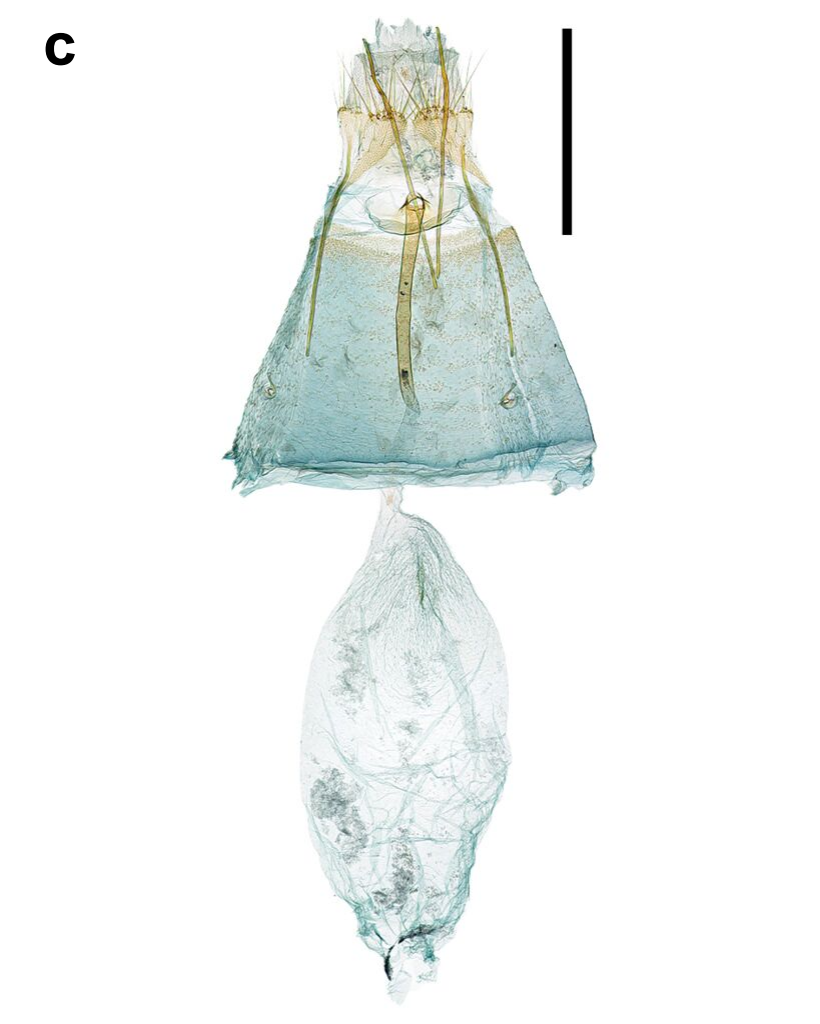
*A. koreana* sp. nov. (♀, Holotype, gen. slide no. HNUSEL-1009).

**Table 1.** Specimens and barcode numbers used in the analysis.

No.	Species name	Voucher number	Accession number (GenBank)	COI region	Depositor	Locality
1	*Acrolepiopsis sapporensis*	HNU_DNA_9588	PV746214	658bp	HNUSEL	Korea
2	*Acrolepiopsis sapporensis*	HNU_DNA_9589	PV748134	658bp	HNUSEL	Korea
3	*Acrolepiopsis sapporensis*	HNU_DNA_9591	PV746219	658bp	HNUSEL	Korea
4	*Acrolepiopsis sapporensis*	HNU_DNA_10826	PV748137	658bp	HNUSEL	Korea
5	*Acrolepiopsis sapporensis*	HNU_DNA_10828	PV746237	658bp	HNUSEL	Korea
6	*Acrolepiopsis sapporensis*	HNU_DNA_10841	PV748136	658bp	HNUSEL	Korea
7	*Acrolepiopsis koreana*	HNU_DNA_10839	PV748135	658bp	NIBR	Korea
8	*Acrolepiopsis quinquelobatae*	HNU_DNA_10048	PV748139	658bp	HNUSEL	Korea
9	*Acrolepiopsis quinquelobatae*	HNU_DNA_10049	PV748140	658bp	NIBR	Korea
10	*Acrolepiopsis quinquelobatae*	HNU_DNA_10050	PV748138	658bp	HNUSEL	Korea
11	*Acrolepiopsis quinquelobatae*	HNU_DNA_10051	PV748141	658bp	HNUSEL	Korea
12	*Acrolepiopsis quinquelobatae*	HNU_DNA_10052	PV748142	658bp	HNUSEL	Korea
13	*Acrolepiopsis japonica*	HNU_DNA_9614	PV748143	658bp	HNUSEL	Korea
14	*Acrolepiopsis nagaimo*	HNU_DNA_9609	PV748145	658bp	HNUSEL	Korea
15	*Acrolepiopsis nagaimo*	HNU_DNA_9611	PV748144	658bp	HNUSEL	Korea
16	*Acrolepiopsis nagaimo*	HNU_DNA_9612	PV748147	658bp	HNUSEL	Korea
17	*Acrolepiopsis nagaimo*	HNU_DNA_10829	PV748149	658bp	HNUSEL	Korea
18	*Acrolepiopsis nagaimo*	HNU_DNA_10830	PV748146	658bp	HNUSEL	Korea
19	*Acrolepiopsis nagaimo*	HNU_DNA_10831	PV748148	658bp	HNUSEL	Korea
20	*Glyphipterix forsterella*	HNU_DNA_5985	PV746250	658bp	HNUSEL	Korea
21	*Glyphipterix forsterella*	HNU_DNA_5986	PV746254	658bp	HNUSEL	Korea
